# Influence of Visual Coding Based on Attraction Effect on Human–Computer Interface

**DOI:** 10.3390/jemr18020012

**Published:** 2025-04-08

**Authors:** Linlin Wang, Yujie Liu, Xinyi Tang, Chengqi Xue, Haiyan Wang

**Affiliations:** Department of Mechanical Engineering, Southeast University, Nanjing 211189, China; linlinwang@seu.edu.cn (L.W.); yliu319@changan-mazda.com.cn (Y.L.); xinyi_tang@seu.edu.cn (X.T.); whaiyan@seu.edu.cn (H.W.)

**Keywords:** attraction effect, information coding, eye movement, visual cognition, decision-making

## Abstract

Decision-making is often influenced by contextual information on the human–computer interface (HCI), with the attraction effect being a common situational effect in digital nudging. To address the role of visual cognition and coding in the HCI based on the attraction effect, this research takes online websites as experimental scenarios and demonstrates how the coding modes and attributes influence the attraction effect. The results show that similarity-based attributes enhance the attraction effect, whereas difference-based attributes do not modulate its intensity, suggesting that the influence of the relationship driven by coding modes is weaker than that of coding attributes. Additionally, variations in the strength of the attraction effect are observed across different coding modes under the coding attribute of similarity, with color coding having the strongest effect, followed by size, and labels showing the weakest effect. This research analyzes the stimulating conditions of the attraction effect and provides new insights for exploring the relationship between cognition and visual characterization through the attraction effect at the HCI. Furthermore, our findings can help apply the attraction effect more effectively and assist users in making more reasonable decisions.

## 1. Introduction

The development of information technology with big data, artificial intelligence (AI), and augmented reality (AR) technology as the core is becoming increasingly advanced. The technological form of human society is changing from informatization to intelligentization. Complex information systems transform massive multidimensional data into accurate and effective information, which is finally presented to decision-makers through the human–computer interface (HCI). In the communication between humans and computers through the HCI, the human cognitive process often uses a simplification strategy, heuristics, which is not always accurate or rational. In an ideal situation, the accurate presentation of information by the HCI can help improve the quality of decision-making [1]. However, due to the limited cognitive ability of humans, heuristics easily lead to irrational decisions and eventually produce decision-making bias. The decision-making bias leads to the inefficiency of the human–computer interaction and the imbalance of human–computer communication, resulting in a reduction in system ergonomics and economic loss [2]. Therefore, studying the visualization of the HCI to explore the characteristics of human visual cognitive bias is an essential way to control human–computer interaction.

As a classical cognitive bias, the attraction effect has attracted extensive attention from experts in decision science, cognitive psychology, consumer behavior, and visual science due to its violation of classical rational choice theory. From the perspective of digital nudging, attraction effects can help architects change humans’ choices in predictable ways [3], but the robustness of their applications in areas such as health management, online shopping, and social media has been controversial. Therefore, this research aims to combine the visual elements of the HCI and attraction effect to explore the stability of the attraction effect in the HCI and how the visual elements affect the strength of the effect.

### 1.1. Analysis of Attraction Effect and Properties

In the cognitive process, decisions made by humans are dominated by contextual information. Contextual effects such as the attraction effect, compromise effect, and similarity effect and their relationships have been explored. The attraction effect is also known as the decoy effect or asymmetric dominance effect. Moreover, when facing alternatives I and II of positive and negative properties, respectively, the probability of the user choosing I increases upon introducing alternative III, which is similar but weaker than alternative I’s positive property. The application of the attraction effect in decision-making refers to changing the choice probability between two original alternatives by introducing a Decoy. The one with increasing probability is the Target, and the one with which it is competing is the Competitor. Recent eye tracking studies have confirmed that such contextual effects are closely associated with visual attention dynamics. For instance, **one study** reviewed extensive empirical evidence, showing that gaze patterns can predict choice outcomes [4]. Contextual cues influence attentional allocation, thereby modifying decision strength [5]. Similarly, **Noguchi and Stewart (2018)** provided evidence that participants dynamically sample information across alternatives based on contextual relevance, as reflected in their eye movements [6]. These findings suggest that the attraction effect is not only a result of value comparison but also closely tied to how visual information is processed during decision-making.

**Figure 1:**
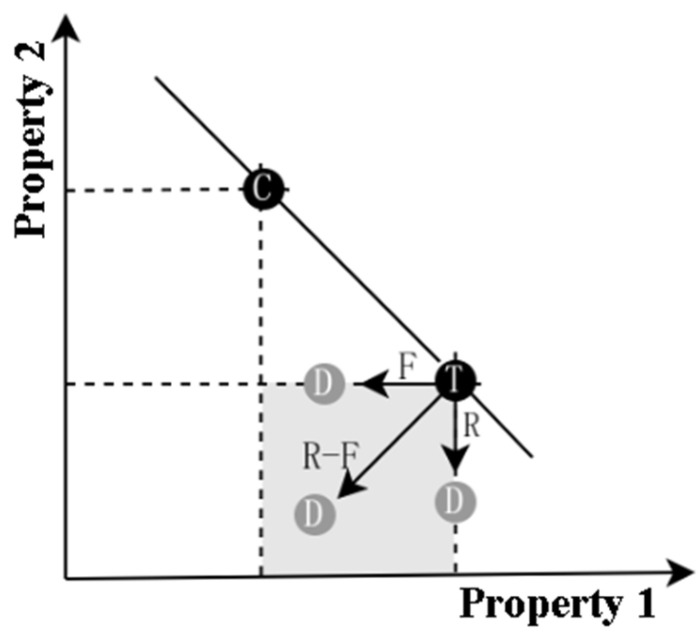
The alternative property space (the shadow area represents the range of property values for D governed by T. Decoy: D, Target: T, Competitor: C).

Preconditions for the attraction effect to be stimulated as follows: a Decoy must exist and the Target and Competitor must present at least two properties [7]. The Target and Competitor must each have a relative positive property to ensure comparability between them. The attraction effect strength of the Decoy is different in different positions of the alternative property space. As shown in Figure 1, the direction of the Decoy with the same value as the Target’s positive property is range (R), the direction of the Decoy with the same value as the Target’s negative property is frequency (F), and the direction of the Decoy weaker than the Target’s two property values is range–frequency (R–F) [8]. Attraction effects can be generated in both R and R–F directions. The attraction effect in the direction of F is not significant [9].

### 1.2. Application of Attraction Effect in HCI and Eye Movement Technology

With technological innovation, research into decision bias in the HCI of complex information systems has gradually attracted attention [10]. In online network context-related research, the attraction effect has been used as a means of digital nudging [3], which supports the prospect of applying the attractiveness effect to HCIs in fields such as multi-criteria decision analysis [11], behavioral economics [12], choice architecture [13], decision support systems [14] and recommender systems [15,16] to exploit decision bias, but there has been controversy about its robustness. Some studies believe that the attraction effect only exists in a digital display, and the image presentation, the change in the presentation order of the alternatives, and the economic impact will all lead to the weakening or even disappearance of the attraction effect [17,18]. Other studies [19,20] show that the strength of the attraction effect depends on the range of alternative property values, and it can be stably stimulated as long as it is within the range. In summary, the attraction effect is affected by many factors, and the information recognition of the online environment needs to be transformed into visual elements that are easy to cognize through visualization technology and finally presented to the decision-makers on the HCI [21,22]. In this context, vision is the main information channel and eye movement becomes the main physiological measurement method in the application of the HCI. Regarding eye movement, our visual focus stays around 50–70 ms at each point in general [23]. This is enough time for us to acquire most of the information, but if a deeper understanding is required, a longer time may be required, with usually 100–200 ms recorded as one fixation [24]. It can be used to analyze which elements in the interface can better attract users’ attention caused by the attraction effect and then optimize the interface design. This supports the existence of a potential influence of visual characteristics of HCI elements on the attraction effect.

In economic consumption decision-making, the assumption of complete rationality entails that users can objectively compare alternatives and find out the one with maximizes benefits when making decisions. However, in real-life applications, such as the wise information technology of med (WITMED) [25], intelligent manufacturing (IM) [26], nuclear power plants (NPPs), electronic centralized aircraft monitoring (ECAM), manned spacecraft, and intelligent transport systems (ITS) [27] from the perspective of the socio-cognitive approach [28], decision-making cannot be carried out with complete rationality. When faced with different situations, especially a large amount of information, due to the limitations of their cognitive ability, users may keep information needs within their cognitive ability and formulate cognitive shortcuts called heuristic strategies [29]. Heuristics are useful when tasks require them to focus on specific types of information or to make quick decisions and analyses [30]. But in some cases, they can lead users becoming lost and producing cognitive biases. Many theoretical models have explained attraction effects, including Multi-Attribute Decision Field Theory (MADFT) [31], Two-Stage Process [32], Significance Theory [33], etc. Trueblood et al. summarized and compared the theoretical mechanisms of different models and found that many models included attention [34], indicating the importance of attention to the attraction effect. The common foundation of the above theories is that when users are faced with a variety of choices with different properties, their attention cannot be allocated to all the details. Instead, attention is focused on a subset and the individual accumulates evidence about the information needed for the current task [34]. In the application of the attraction effect, the similarities and differences between the Decoys and Targets make the attention focus on the comparison of the above two alternatives [7] and affect the user’s final decision through the interface, which is beneficial for the national economy and people’s livelihood.

### 1.3. Analysis of Visual Element of Attraction Effect on HCI

The elements of the HCI include color, layout, icon, and structure (referred to as coding mode in this research), and there are various properties of the attraction effect (used in the coding attribute in this research). When analyzing the role of the attraction effect in the HCI, during the pre-attentive stage, Gestalt principles such as the proximity principle guide visual cognitive strategies by facilitating the processing of visual information. Specifically, the attraction effect primarily influences the preliminary processing and organization of information by enhancing the significance of certain visual characteristics. In the selective attention stage, Gestalt principles such as the similarity principle guide visual cognitive strategies that affect the distribution and preference of attention, thereby playing a crucial role in the final decision-making. The interaction of these two stages makes the attraction effect play a key role in guiding and shaping attention during visual cognition.

It has been shown that the information features shared by the Target and Decoy enhance the attraction effect. For example, Bhatia S’s study found that the strength of the attraction effect increases when users pay more attention to the shared features of the Target and Decoy [35]. Therefore, this research conjectures that if the Decoy and Target are displayed in the same coding mode on the HCI, and the Competitor is different from them, the Decoy and Target will be given prioritized attention in the pre-attention stage because the proximity principle dominates the information feature grouping, and the attribute shared between them will be enhanced in the selective attention stage. Attention is more focused on these two alternatives, and the attraction effect is enhanced. If the Target and Competitor are displayed in the same coding mode, the equal status of the Target and Competitor will be preferentially noticed, which means that the visual recognition of the relationship between the Target and Decoy will be weakened, and the attraction effect will be weakened to a certain extent. Therefore, the purpose of this research is to explore the role of different coding modes and attributes in the variation in attraction effect strength. Based on previous research on the attraction effect, we choose visual coding as the research element: taking online shopping as the research scenario, we explore the effect of different coding modes of HCI elements and different coding attributes on the strength changing in the attraction effect. HCI elements refer to coding modes while similarity and difference between the Targets and Decoys refer to coding attributes, and the above analysis about visual cognition and the attraction effect based on attention changes leads us to the following hypotheses:

**Hypothesis** **1:** *Different coding attributes will change the strength of the attraction effect. The attraction effect will be enhanced under the similarity attribute and will be weakened under the difference attribute.*

**Hypothesis** **2:** *There is an interaction between the coding mode and coding attribute. The effect of coding attributes on the attraction effect is affected by different coding modes.*

**Hypothesis** **3:** *Different coding modes may affect the intensity of the attraction effect by changing attention, and different coding attributes may affect the intensity of the attraction effect by changing the direction of attention change.*

## 2. Materials and Methods

### 2.1. Participants

The participants comprise 65 undergraduate and graduate students from Southeast University with a large amount of online shopping experience and strong computer operation abilities, all of whom are right-handed (29 males and 36 females and aged between 23 and 27 years old). The participants have normal vision or corrected vision and no color blindness or color weakness. Each of them was provided written informed consent forms approved by the Ethics Committee of Southeast University affiliated with Zhongda Hospital. Before this experiment, all participants did not participate in the pre-experiment and were not familiar with this experiment.

### 2.2. Experiment Environment

The experiment was conducted in the Human Factors Engineering Laboratory of Southeast University. The experimental environment ensured that the participants completed the experiment without interference. The experimental program was presented to the participants on a 24-inch interface (2K resolution, brightness: 92 cd/cm^2^). The participants maintained a viewing distance between 600 mm and 650 mm from the interface. The laboratory was illuminated by four 40 W fluorescent lamps, ensuring a normal lighting environment. The decision-making process of the shopping interface as the experimental scenario is affected by product appearance, preference, time, etc. To reduce the influence of irrelevant variables, this research designed the following virtual shopping scenario. Each alternative contains the following four pieces of information: product serial number, product image, product category, and two properties of the product.

(1) Product images: To preserve the situational context of the image while reducing its interference with user decision-making, all alternatives use the same product image.
(2) Product categories: To reduce the impact of product appearance on decision-making, this research selected practical products to ensure that the products are familiar to the majority of users. Finally, eight categories are determined, including cameras, headphones, electric toothbrushes, humidifiers, suitcases, projectors, robots, and bicycles.
(3) Product properties: The selection of product properties combines the two methods of a questionnaire and pre-test. Regarding the questionnaire, the results are derived from the survey results of 37 valid questionnaires. They compare the weights of product properties involved in purchasing products and the selectable properties are derived from the product details of actual shopping websites. Each product in the questionnaire includes at least five properties for users to choose from. Regarding the pre-test, this research set up a binary set containing the Targets and the Competitors and a ternary set with the Decoys added. The questionnaire participants were asked to conduct the pre-test and were required to select the product they most wanted to buy based on the comprehensive judgment of the product’s properties. The negative properties value of the Competitor’s scheme is 10% weaker than the negative properties value of its dominant scheme, and the positive property value is the same as its dominant scheme. The properties values of the alternatives are adjusted according to the pre-test results to ensure that the experimental materials meet the preconditions for the attraction effect to be stimulated, as seen in Figure 1, especially if the properties of the Competitors are within the range threshold of the stimulated attraction effect. Information on the experiment products is shown in Table 1.

### 2.3. Experiment Apparatus

The devices used in this experiment are Tobii X2-30 and a desktop computer.

(1) Tobii X2-30 is a compact device placed under a desktop computer interface that records participants’ eye movement data on the desktop computer interface. The non-contact sampling method and compensation algorithm are used to sample the position of participants’ eyeballs at a frequency of 30 Hz. Participants are free to move their heads in a free range without wearing any device, and the device does not interfere with participants’ visual activities. The experiment is programmed, stimuli presented, and eye movement data statistics performed using Tobii Studio 3.2.3.
(2) The desktop computer interface, which is connected to eye movement, displays the experimental materials. The experimental materials used as stimuli are displayed on a 27-inch display screen (617.50 mm × 371.20 mm) with a resolution of 3840 × 2160 and a brightness of 92 cd/cm^2^.

### 2.4. Materials

**Figure 2:**
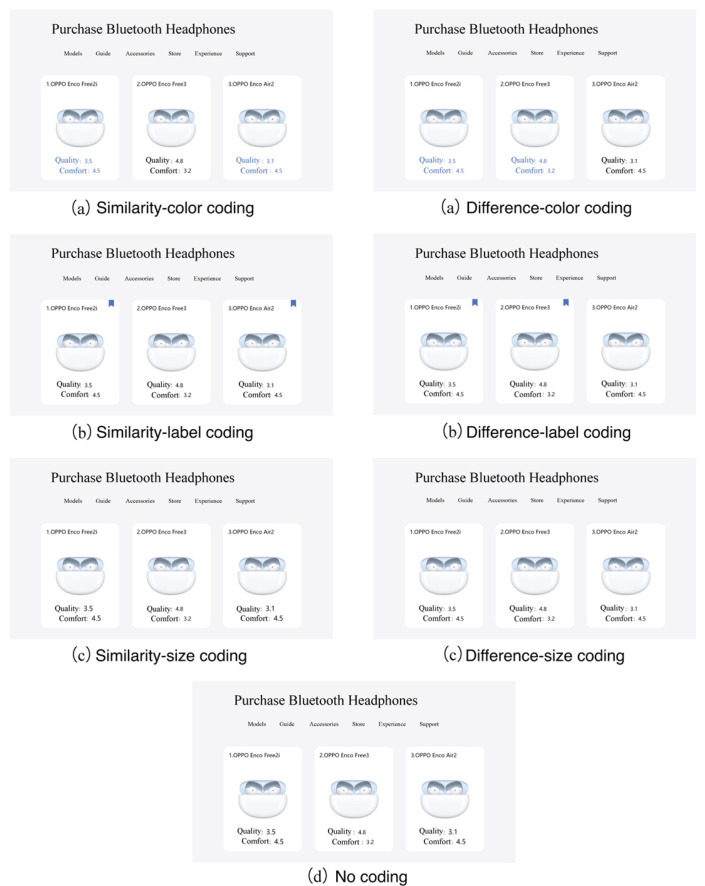
Coding attributes and coding modes of the experimental materials (taking headphones as an example).

To reduce the impact of product appearance on decision-making as much as possible, eight common product categories were selected and the sample size of the participants was expanded to 65. The independent variables include two categories: coding attributes and coding modes. Coding attributes entail similarity coding or difference coding. The former refers to the use of the same coding for Targets and Decoys (equivalent to enhancing the similarity between Targets and Decoys), and the latter refers to the use of the same coding for Targets and Competitors (equivalent to enhancing the difference between Targets and Decoys). Coding modes include color, shape, label, and size. They need to be presented to users with the help of the HCI, while shape changes the presentation carrier of the HCI and is therefore excluded. Taking headphones as an example, the combination of coding attributes and coding modes of the experimental materials is shown in Figure 2. The dependent variable is the participants’ choice of purchase products and performance in the decision-making process under the influence of different coding attributes and coding modes.

### 2.5. Experimental Setting

Different decision-making biases have different causes, so different research paradigms are used to meet the bias-inducing conditions. The attraction effect leads to changes in the probability of selecting alternatives, meaning the introduction of a Decoy increases the probability of selecting the Target. Therefore, the standard experimental paradigm is adopted, which involves forced-choice decisions between multiple alternatives to compare the magnitude of probability changes across the alternatives. In terms of experimental design, some researchers adopt a between-subjects design to avoid the influence of memory effects on the results. Similarly, given the potential impact of individual differences on the experiment, some researchers use a within-subjects design. Therefore, taking the above considerations into account, the experiment was designed as follows.

The formal experiment was set up as a three-factor within-subject design of 2 (binary set “Competitor + Target”; ternary set “Competitor + Target + Decoy”) × 4 (coding modes: color; label; size; and no-coding mode) × 2 (coding attributes: similarity coding and difference coding). Since each participant could not encode the same product in different ways, all participants were divided into four groups (every two products appeared in one coding attribute/mode). The product categories selected were cameras, headphones, electric toothbrushes, humidifiers, suitcases, projectors, robots, and bicycles. Each product included five non-repeating binary sets and 10 corresponding ternary sets. Each coding mode contains five ternary sets with difference coding and five ternary sets with similarity coding; the ternary sets with difference encoding and similarity encoding with the no-coding mode are the same. Therefore, there are 40(=8×5) trials for binary sets and 70 (=5×(2+6×2)) trials for ternary sets. To safeguard the experimental purpose, half of the total tests were designated as filler tests in both the binary and ternary sets. Participants in the binary sets completed a total of 60 tests, while those in the ternary group completed 105 tests in total.

To ensure an adequate sample size for detecting statistically significant effects in our within-subject design, we conducted an a priori power analysis using G*Power. For our three-factor within-subject design (2 × 4 levels), we assumed a medium effect size (f = 0.25), a significance level (α = 0.05), and a desired statistical power of 0.80. Given that this is a within-subject design, we set 1 group and 8 measurements (reflecting the 2 × 4-factor structure). We also set the correlation among repeated measures to 0.5 and the nonsphericity correction ε to 1. The power analysis indicated that a minimum total sample size of 16 participants would be required to achieve the desired power level of 0.80 (actual power = 0.8198). Our study included 65 participants, which exceeded this minimum requirement, ensuring that our sample size was sufficient to detect medium-sized effects within our experimental design.

### 2.6. Procedure

Before the formal experiment, the experimenter gave each participant a detailed explanation and training, introducing the shopping scenario of each product. The shopping scenario provided participants with a hypothetical purchase premise and explained the upcoming properties of products to ensure that each participant was familiar with their tasks.

After the formal experiment began, the participants first performed a ternary set selection purchase task. In each trial, the participants were shown the Decoy, Target, and Competitor of the same product and were asked to select the product they wanted to buy most based on the two given properties. Each product was marked with a serial number, and the participants were asked to press the number button corresponding to the serial number of the product they wanted to buy. After the participant pressed the button, the program automatically recorded the answer and entered the next trial. The experimental program set a rest prompt. At the midpoint of the experimental session, participants were given the option to take a break or continue based on their own judgment in order to minimize fatigue. The ternary set tasks were completed in about 20 min.

**Figure 3:**
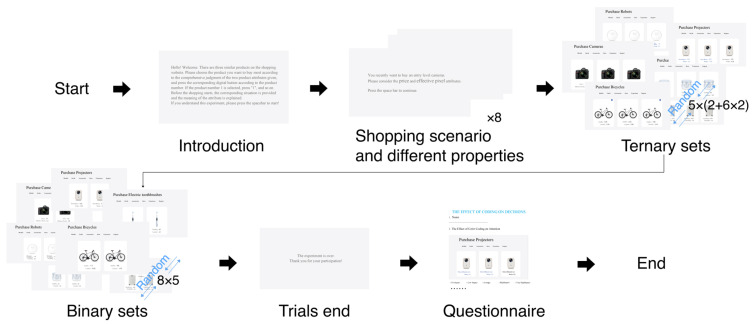
Experiment procedures.

In the formal experiment, tasks in the binary set required participants to choose between the Target and the Competitor. The experimental procedure followed the same steps as previously outlined, with tasks completed in approximately 10 min. To mitigate potential learning effects, the binary and ternary set experiments were conducted at least one day apart. After completing the experiment, participants were asked to fill out a questionnaire regarding attention to different coding modes. The questionnaire gathered data on the influence of various coding modes on participants’ attention and their preferences. The experiment procedures are shown in Figure 3.

### 2.7. Data Analysis

Participants who selected the Decoy alternatives were excluded from this research. Two participants were excluded due to poor performance, and the Relative Selection Share of the Target (RST) for all remaining participants across the binary and triad sets, under different coding attributes and modes was calculated to support Hypotheses 1 and 2.

Additionally, this study analyzed the gaze plot (GP) [36], heat map (HM), and relative fixation time (RFT) to support Hypothesis 3. Following the data collection process for eye movements, 25 participants with a collection rate below 70% were excluded based on the behavioral data. Consequently, we present an analysis of the eye movement data from a total of 38 participants. SPSS 26 is used for data processing and charting.

## 3. Results

### 3.1. Behavioral Data

Statistical methods were employed to further investigate the impact of the experimental material layout on the attraction effect. The RST for each participant under different conditions was calculated for the normality test. As the sample size under different conditions exceeded 50, the Kolmogorov–Smirnov test was employed. The results indicated that the experimental data (*p* < 0.05) did not follow a normal distribution. The histogram and QQ plot of the observed data displayed significant deviations, with skewness and kurtosis having absolute values greater than 1.96. Given the within-subject design of the experiment, this study referred to the non-parametric test method proposed by Marini M et al. [7] and employed the related-samples non-parametric test in SPSS to analyze the data from both horizontal and vertical perspectives. As a result, the influence of the alternative layout was controlled for, with the primary focus placed on the analysis of independent variables.

#### 3.1.1. RST for Coding Modes and Attributes

**Figure 4:**
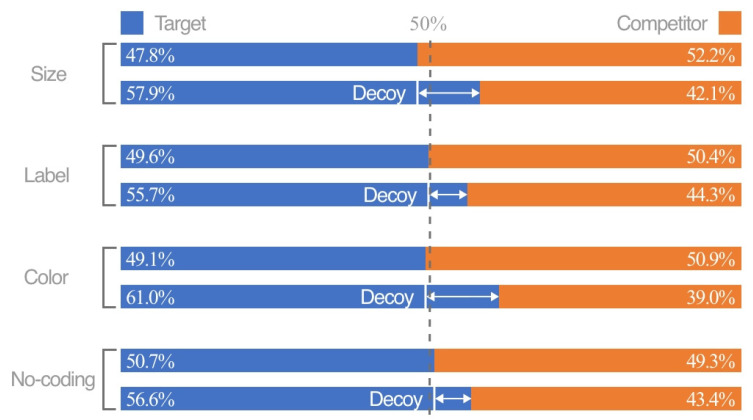
The RST of coding modes, respectively (the first line of each coding mode is a binary set, and the second line is a ternary set).

**Figure 5:**
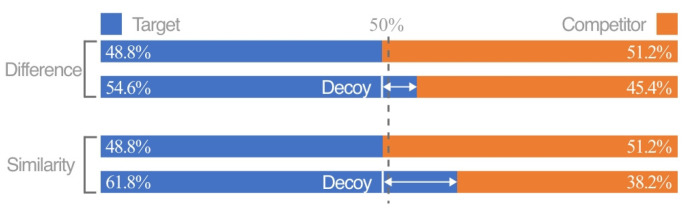
The RST of coding attributes, respectively (the first line of each coding mode is a binary set, and the second line is a ternary set).

For the coding modes illustrated in Figure 4, the RST of the no-coding group, which serves as the baseline for comparison with other coding modes, increases by 5.9%. The RST for both color coding and size coding showed significant increases of 11.9% and 10.1%, respectively. The difference in the RST between the label-coding group and the no-coding group was marginal, at 6.1%. Regarding coding attributes, as demonstrated in Figure 5, the RST for similarity coding (13.0%) is significantly higher compared to that for difference coding (5.8%).

#### 3.1.2. Horizontal Analysis for Single Coding Mode and Attribute

**Figure 6:**
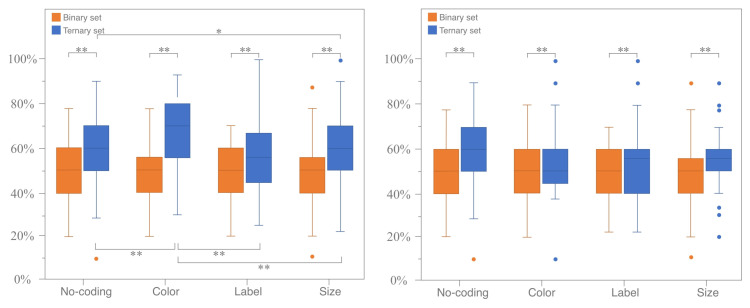
The significance test for the RST distribution map under the coding attributes of similarity (**left**) and difference (**right**). (* means *p* < 0.05 and ** means *p* < 0.01).

The RST of each participant’s ternary and binary sets under a single coding mode and attribute was calculated. The Wilcoxon signed-rank test for paired samples was used to analyze the differences. The test results are shown in Figure 6. Under the coding attribute of similarity, the RST for the ternary sets of color coding (*p* < 0.05, Z =−5.564, r ≈−0.701), label coding (*p* = 0.008 < 0.05, Z =−2.646, r ≈−0.333), and size coding (*p* < 0.00, Z =−4.129, r ≈−0.520) is significantly greater than that of the binary sets, indicating that the attraction effect differs and remains stable across different coding modes. Under the coding attribute of difference, the RST of ternary sets encoded by color (*p* = 0.006 < 0.05, Z =−2.731,r≈−0.344), label (*p* = 0.037 < 0.05, Z =−2.090,r≈−0.263), and size (*p* = 0.006 < 0.05, Z =−2.761,r≈−0.348) is also significantly greater than that of binary sets. Additionally, a significant difference in the RST between ternary and binary sets is observed in the no-coding mode (*p* = 0.007 < 0.05, Z =−2.675,r≈−0.337). No significant difference between the similarity and difference coding attributes was found in the no-coding mode, which only serves as a comparison benchmark for different coding modes and attributes. The data are non-normally distributed; therefore, non-parametric tests are used.

#### 3.1.3. Vertical Analysis for Comparing Different Coding Modes and Attributes

**Figure 7:**
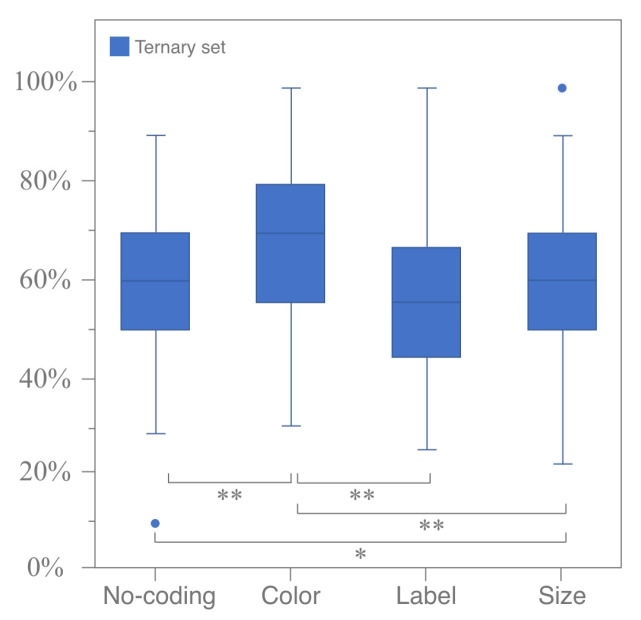
Significance test of RST for all coding modes under coding attribute of similarity (ternary sets only). (* means *p* < 0.05 and ** means *p* < 0.01).

The horizontal analysis results demonstrated that different combinations of coding modes and attributes can evoke attraction effects. This section compares the intensity of attraction effects induced by different coding modes from vertical perspectives. For various coding modes, the Friedman test for k-related samples was used to examine differences in the RST of ternary sets under the same attribute. The results indicated no significant difference in the RST across all coding modes under the coding attribute of difference (*p* = 0.888 > 0.05), thus partially supporting Hypothesis 1. However, there was a significant difference in the RST across all coding modes under the coding attribute of similarity. A further Wilcoxon signed-rank test of paired samples was conducted to compare coding modes pairwise under the coding attribute of similarity. It was found that significant differences exist between color coding and label coding (*p* < 0.05, Z =−3.537, r ≈−0.446), size coding (*p* = 0.017 < 0.05, Z =−2.385, r ≈−0.300), and the no-coding mode (*p* = 0.001 < 0.05, Z =−3.420, r ≈−0.431). There was a marginally significant difference between size coding and the no-coding mode (*p* = 0.055 < 0.1, Z =−1.920, r ≈−0.242) but no significant difference between size coding and label coding (*p* = 0.105 > 0.05, Z =−1.621, r ≈−0.204). Similarly, no significant difference was found between label coding and the no-coding mode (*p* = 0.982 > 0.05, Z =−0.022, r ≈−0.003). These results, as illustrated in Figure 7, confirm Hypothesis 2.

**Figure 8:**
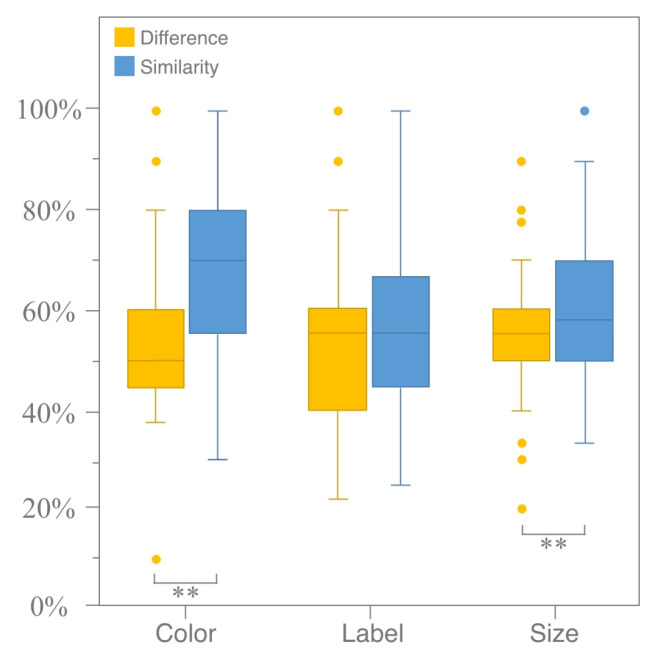
Significance test of RST in same coding mode under different coding attributes (ternary sets only). (** means *p* < 0.01).

For different coding attributes, the Wilcoxon signed-rank test for paired samples was employed to analyze the differences in the RST of ternary sets within the same coding mode. A significant difference in the RST was observed between different coding attributes under color coding (*p* < 0.05, Z =−4.079, r ≈−0.514) and size coding (*p* = 0.023 < 0.05, Z =−2.276, r ≈−0.287). However, no significant difference in the RST was found between different coding attributes under label coding (*p* = 0.314 > 0.05, Z =−1.006, r ≈−0.127), as illustrated in Figure 8.

### 3.2. Eye Movement Data

#### 3.2.1. Gaze Plot and Heat Map

**Figure 9:**
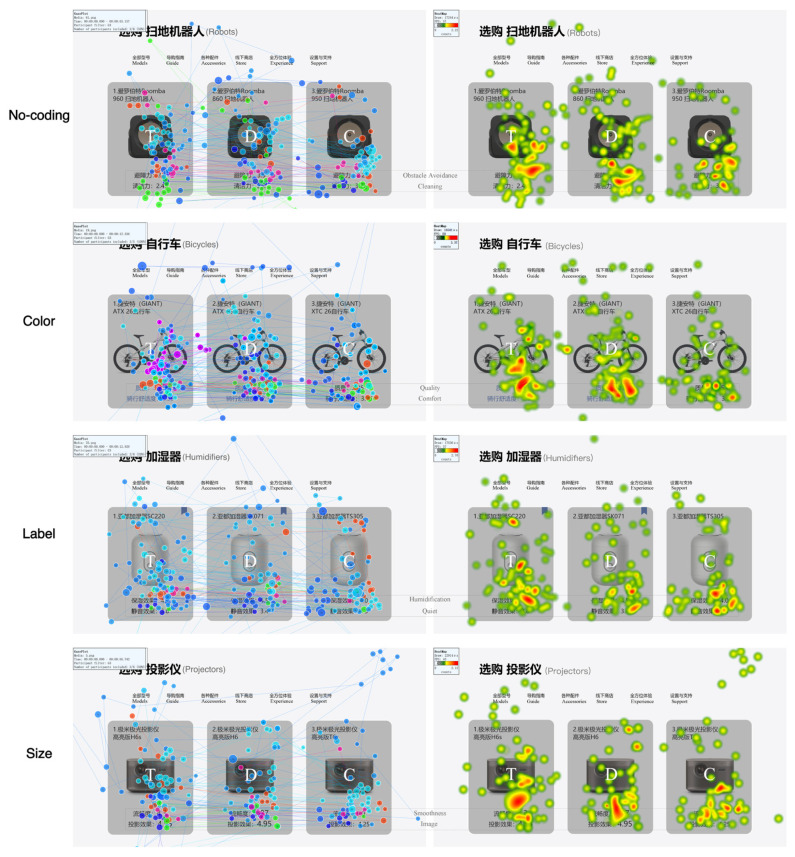
GP and HM with different coding modes under coding attribute of similarity.

This study superimposed the GP and HM for a single group of participants [37], specifically within the ternary set. As there were no significant differences across different coding modes under the attribute of difference, we limited our comparison of the GP and HM to different coding modes under the attribute of similarity, as illustrated in Figure 9. The results showed that when the no-coding mode was applied, participants predominantly focused on the Target and Decoy. Under color coding, participants spent more time focusing on the Target and Decoy, while the gaze time on the Competitor was relatively reduced compared to the no-coding condition. When comparing color coding to label coding, participants allocated more attention to the Target and Decoy under color coding. However, there was no significant difference in attention distribution between color coding and size coding. Label coding resulted in a more balanced distribution of attention compared to other coding modes. The attention distribution under size coding was similar to that in the no-coding mode, but it was difficult to visually differentiate the changes in the proportion of attention toward the Competitor between these two modes. Compared to size and label coding, the proportion of attention directed toward the Target and Decoy was greater.

An analysis of the visualized eye movement data indicated that, of the three coding modes, color coding resulted in the most significant changes in attention compared to no coding. Size coding showed potential differences, while label coding had little effect and, in some cases, even counteracted attention shifts.

**Figure 10:**
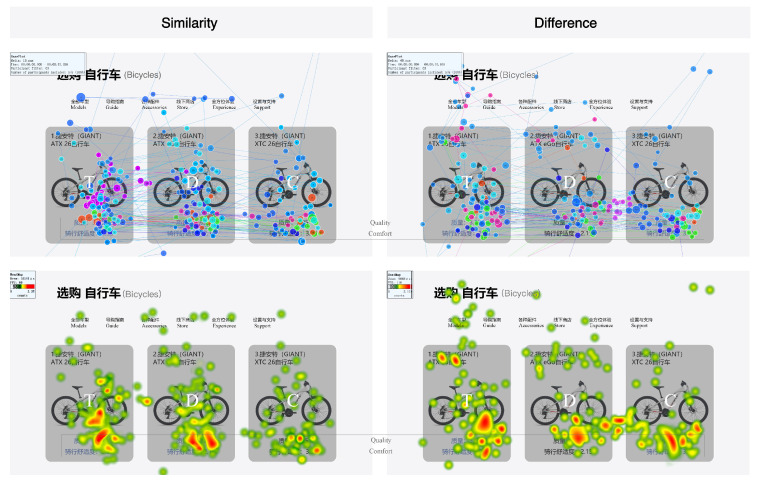
GP and HM with different coding attributes under color coding.

**Figure 11:**
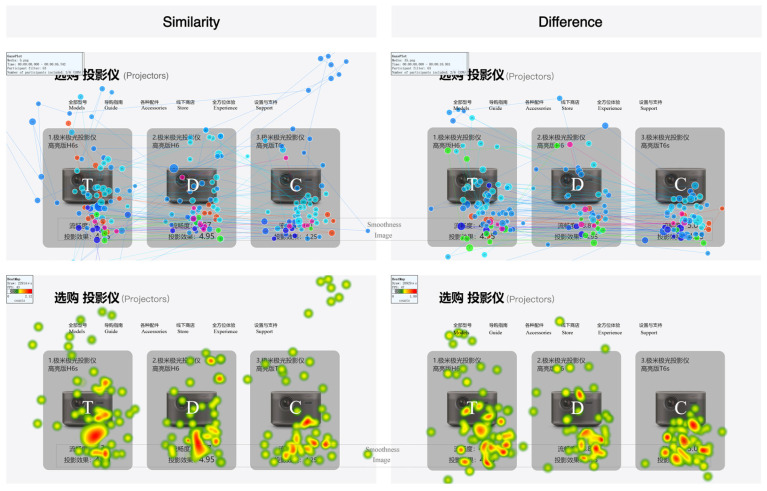
GP and HM with different coding attributes under size coding.

When comparing the GP and HM under a single coding mode, both similarities and differences were observed. Under the coding attribute of similarity, color coding enhanced the relational attributes between the Target and Decoy, focusing participants’ attention on these two alternatives. In contrast, under the coding attribute of difference, while the relational attributes between the Target and Decoy remained, color coding highlighted the competitive relationship between the Target and the Competitor, significantly increasing participants’ attention to the Competitor (see Figure 10). For size coding, the changes in attention across different coding attributes were similar to those observed under color coding (see Figure 11).

**Figure 12:**
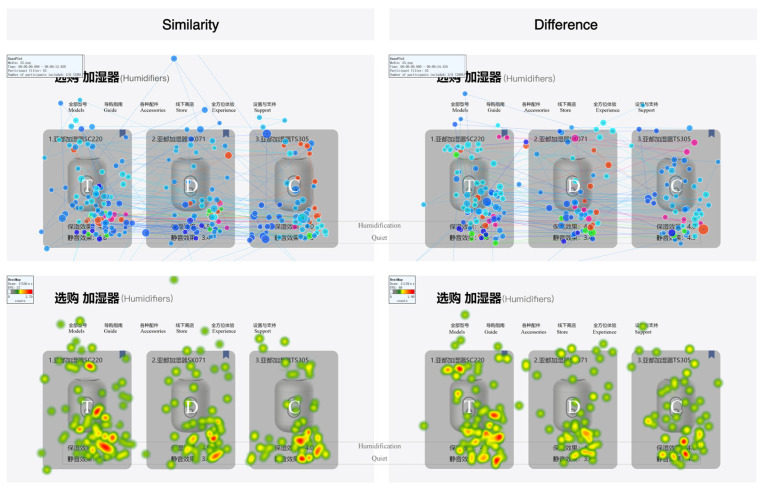
GP and HM with different coding attributes under label coding.

Under label coding, participants’ attention to the Target did not show significant changes as the coding attributes varied. Similarly, the Competitor did not receive more attention from participants due to being label-encoded (see Figure 12). This also indicates that participants rarely noticed the presence of labels during the search process, which might explain why their attention remained unchanged.

#### 3.2.2. Relative Fixation Time

RFT in this research is defined as the proportion or percentage of time participants spend fixating on a specific area or target relative to the total fixation time. In the ternary set, each alternative on a single test material was designated as an area of interest (AOI) to collect participants’ fixation time (FT) on the Target and Competitor. The RFT for the Target was then calculated. The RFT for each condition was averaged across all test materials for each participant, resulting in 8(=4 codingmodes×2 codingattributes) groups of data. Normality tests were conducted on the data, and for groups that did not meet the normal distribution criteria, non-parametric tests were performed using SPSS. The primary aim of the data analysis was to compare differences in attention allocation to the Target across different coding modes or attributes. Therefore, the data were analyzed vertically, similar to the behavioral data.

**Figure 13:**
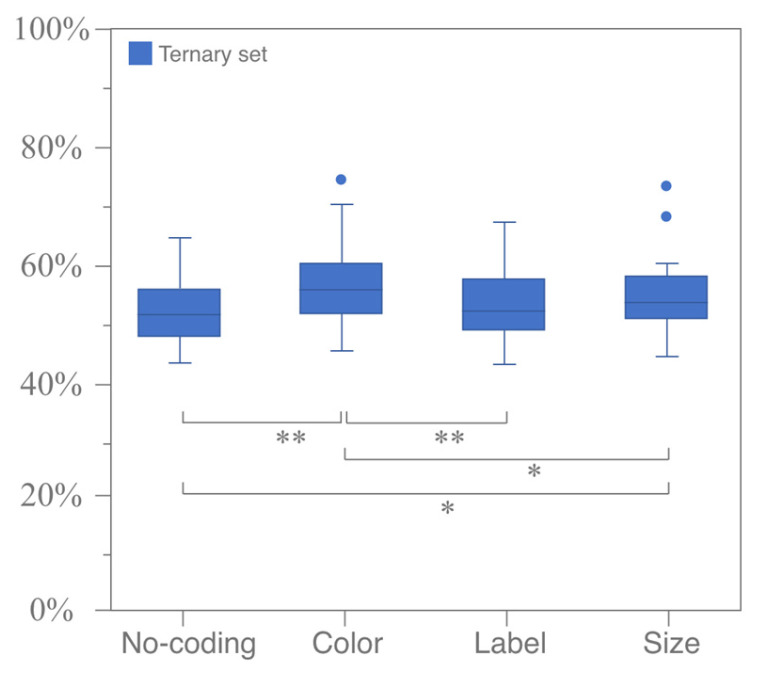
Significance test of RFT of different coding modes under coding attribute of similarity. (* means *p* < 0.05 and ** means *p* < 0.01).

**Figure 14:**
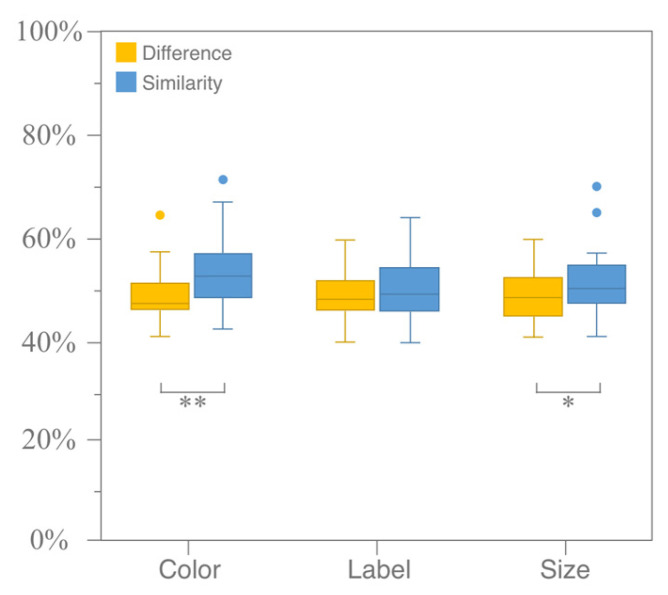
Significance test of RFT of different coding attributes under three coding modes. (* means *p* < 0.05 and ** means *p* < 0.01).

(1) Coding modes
  The Friedman test for k-related samples was used to analyze the RFT under the coding attribute of similarity across different coding modes, revealing a significant difference (*p* = 0.015 < 0.05, $\chi^{2}$ = 10.421). A further pairwise comparison using the Wilcoxon signed-rank test showed significant differences between color coding and the no-coding mode (*p* = 0.003 < 0.05, Z =−2.922, r ≈−0.474) and between color coding and label coding (*p* = 0.007 < 0.05, Z =−2.690, r ≈−0.436). Marginal significance was found between color coding and size coding (*p* = 0.073 < 0.1, Z =−1.791, r ≈−0.291) and between size coding and the no-coding mode (*p* = 0.091 < 0.1). No significant differences were observed in the other pairwise comparisons, as shown in Figure 13. Using the same analytical method, the RFT under the coding attribute of difference across different coding modes was compared, and the results indicated no significant differences between the coding modes (*p* = 0.974 > 0.05, $\chi^{2}$ = 0.221).
(2) Coding attributes
  The Wilcoxon signed-rank test was used to analyze the differences between coding attributes under a single coding mode. The results showed a significant difference in the RFT for the Target only under color coding (*p* = 0.002 < 0.05, Z =−3.154, r ≈−0.512). Marginal significance was observed under size coding (*p* = 0.097 < 0.1, Z =−1.661, r ≈−0.269), while no significant differences were found under label coding, as shown in Figure 14.

### 3.3. Subjective Questionnaire Data

#### 3.3.1. Impact of Attention on Coding Modes

A statistical analysis was conducted on the impact of different coding modes on participants’ attention. A total of 36.92% of participants reported that color coding had a significant impact on their attention, while 49.23% indicated that label coding had no impact at all, and 33.85% felt that size coding had a moderate impact, as shown in Table 2. The five levels of impact were assigned scores ranging from 1 to 5, and the average impact of each coding mode was calculated using the following formula: average impact=(Σ frequency×weight)/total number of participants. The results showed that size coding (2.89) > color coding (2.85) > label coding (1.97), indicating that color and size coding were more attractive to participants, while label coding had relatively low appeal.

#### 3.3.2. Preference Degree for Coding Modes

A ranking of participants’ preferences for different coding modes, along with the reasons for their decisions, was conducted. The average composite score for each alternative was calculated, resulting in the following order: color coding (2.32) > size coding (1.91) > label coding (1.57), as shown in Table 3. Participants indicated that color coding and size coding had a more direct impact on the coding attributes, making them more visually prominent. Some participants also noted that size coding was only noticeable when carefully observed. Label coding, positioned in the upper right corner of the alternatives, was easily overlooked, but the labeled alternatives were perceived as more popular products.

#### 3.3.3. Preference Degree for Coding Alternatives

When answering the question “Do you tend to choose the encoded alternatives during decision-making? (Multiple choices allowed)“, about half of the participants noted that there were certain connections between the encoded alternatives, such as similar overall attribute values or identical values for specific attributes. As a result, they were more inclined to select the encoded alternatives. The other half of the participants insisted that they only considered the attribute values of the products as the basis for their decisions and were not influenced by visual coding. However, behavioral data revealed that certain coding modes significantly increased the strength of the attraction effect under the coding attribute of similarity, suggesting that the latter participants may have exhibited evaluation bias in their responses. A possible explanation is that they were not consciously aware of the influence of coding on the attraction effect.

## 4. Discussion

### 4.1. Theoretical Implications

**Figure 15:**
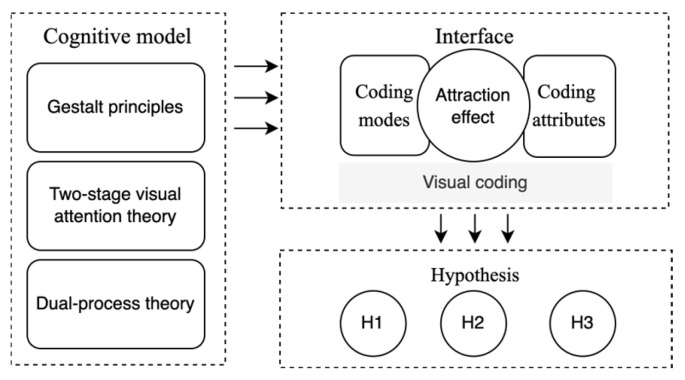
Theoretical relationship in this research.

This research investigates visual elements influencing the attraction effect in HCI. The previous related findings indicated that the Decoy range [19], attribute presentation and order [17], information characterization [21,22], layout of alternatives [38], and decision time [39] can modulate the strength of the attraction effect by attention changing. Specifically, Trueblood et al. highlighted that studying the influence of contextual information on decision-making through information features (i.e., how individuals perceive and store alternatives) is a promising avenue for research [40]. Our results extend those findings by demonstrating the validity of exploring the attraction effect through visual characterization. Similarly, Orquin et al. suggested that visual attention distribution and decision-making regarding alternatives are significantly influenced by information coding, such as position and presentation order, leading to visual cognitive biases [41]. Our findings delve into the visual coding mechanisms underlying the attraction effect, supporting the intrinsic link between visual cognition and value-based preferences (see Figure 15). In the process of exploring the research hypothesis, the following conclusions are summarized:

(1) The impact of different coding modes on the attraction effect: Various coding modes can stimulate attraction effects, but there are differences in intensity. Under the coding attribute of similarity, color coding has the strongest attraction effect, and size coding has a slightly stronger attraction effect than the no-coding mode. There is no significant difference between label coding and size coding. Under the coding attribute of difference, the intensity of the attraction effect produced by each coding mode is roughly the same.
(2) The impact of different coding attributes on the attraction effect: Various coding attributes can stimulate attraction effects, and the intensity depends on the coding mode. In the color and size coding modes, the attraction effect of the coding attribute of similarity is greater than that of difference. Under the label coding mode, there is no significant difference in the intensity of the attraction effect of similarity and difference.

Our findings on gaze patterns and choice behavior align with previous research showing that visual attention significantly contributes to the decoy effect [4,42]. When the stimulus information is processed, the cognitive system will generally perceive the whole first and then perceive the partial characteristics of the stimulus. The introduction of the Decoys changes the overall perception and makes the target seem more prominent and superior. This process is performed through the guidance of attention. Humans regard the object as a whole because of the five important principles of the Gestalt laws: the proximity principle, the similarity principle, the continuity principle, the closure principle, and the common fate principle. These principles form the foundation of visual cognition. For instance, layout coding classifies similar information or distinguishes different information by the relative distance of elements (the proximity principle), and color coding divides information by assigning the same or different colors to information (the similarity principle) [43]. Visual processing occurs in two stages: the pre-attentive stage and the selective attention stage. In the pre-attentive stage, the visual system automatically organizes the world into objects and groups using a visual strategy of parallel search, that is, examining all items with similar features simultaneously. During this phase, users quickly identify basic visual characteristics and filter them automatically, directing their attention to objects with specific characteristics. In other words, an architect can use these features to direct the user’s attention to the object that the architect expects. In the selective attention phase, the visual strategy shifts to a serial search, that is, checking items one by one. As users recognize the relationship between the Decoys and Targets in this process, they will redistribute their attention, thus changing the degree of attention to different alternatives. Furthermore, our findings suggest that different visual attributes may operate at different stages of cognitive processing. Color, as a perceptual feature, may attract early visual attention more effectively than textual labels, thereby influencing decision-making at an earlier, more intuitive processing stage. This explanation aligns with dual-process theories, where visual cues may engage System 1 (fast, automatic processing), while labels require more deliberate System 2 processing [44]. Therefore, the above rules regarding coding modes and attributes can serve as guiding strategies for interface design. At the same time, these findings outline a cognitive model that integrates Gestalt principles, the two stages of visual attention, and dual-process theory, offering a comprehensive explanation of how perceptual features like color influence decision-making more strongly than semantic features such as labels.

### 4.2. Practical Implications

The potential applications of decision-making biases in the HCI have been widely recognized. While prior studies have thoroughly explored the boundary conditions influencing the attraction effect, they have seldom addressed the role of visual display in this context, particularly in the HCI. We summarized the following conclusions based on eye movement data, supplemented by behavioral data and subjective questionnaire data. Specifically, the boundary conditions refer to how different combinations of visual coding modes (e.g., color, label, size) and coding attributes (e.g., similarity, difference) modulate the strength of the attraction effect.

(1) There are differences in the RFT of the Targets for different coding attributes. Specifically, the coding attribute of similarity is greater than that of difference under color coding; the coding attribute of similarity is slightly greater than that of difference under size coding; and there is no difference under label coding. The results of the eye movement data are consistent with the results of the behavioral data. There is no conflict between subjective questionnaires and objective indicators. This indicates that the change in attention caused by coding attributes can affect the intensity of the attraction effect.
(2) There are differences in the RFT of the Targets under the similarity of different coding modes. Specifically, the RFT is the largest under color coding, slightly higher than that of the no-coding mode under size coding, and there is no difference between label coding and the no-coding modes. The results of the eye movement data are consistent with the results of the behavioral data, indicating that color coding has the best effect on attention changing, followed by size coding, and label coding has no obvious effect.

The theoretical findings of the experiment demonstrate that applying coding alternatives based on Gestalt principles can modify the intensity of the attraction effect. However, this modification is observed only when the coding attribute is similarity. Therefore, in practical applications, it is crucial to enhance the visual similarity between the Targets and the Decoys. When this similarity is heightened, the relationship between the two becomes more pronounced, directing attention toward comparing these two alternatives, which ultimately increases the likelihood of the Targets being selected. In contrast, under the coding attribute of difference, the intensity of the attraction effect across the three coding modes does not significantly deviate from the no-coding mode. The eye movement data further supports this, showing no notable difference in participants’ focus on the Targets between coding and no-coding modes. This indicates that the impact of the coding attribute on the Decoy–Target relationship influences user decision-making more substantially than the Competitor–Target comparison emphasized by the coding modes.

When coding attributes are the same, it provides a reference for applying coding modes to influence user decision-making in practical applications. For instance, among the three coding modes, color coding has the most pronounced effect on the attraction effect. Although the eye movement data (including the marginal significance of HM and RFT under each coding mode) and subjective questionnaires indicate that color and size coding have similar impacts on users’ attention, the moderating effect of size coding on the attraction effect remains weaker than that of color coding. This may be related to the different thresholds for various stimuli and the significance of different coding modes. In other words, there may be a correlation between the difference threshold or just noticeable difference and the significance of different coding modes, depending on the context. This study discusses how coding attributes and modes attract attention through website interfaces. This is not only applicable to website interfaces but also to other HCIs.

## 5. Conclusions

In summary, the attraction effect is affected by many factors, and the information recognition of the HCI needs to be transformed into visual elements that are easy to cognize through visualization technology and finally presented to the decision-makers. Eye movement technology can be used to analyze which elements in the interface can better attract users’ attention caused by the attraction effect and then optimize the interface design. Specifically, this research takes a website scenario as an example and compares the enhancement of different coding attributes and modes on the attraction effect, highlighting the distinctions between coding modes and attributes. It not only provides a foundation to empirically support the integration of the attraction effect into HCI system design but also offers theoretical insights into how visual features guide user decision-making.

## Figures and Tables

**Table 1:** Experiment product information.

Product Serial Number	Product Categories	Property 1	Property 2
1	Cameras	Price	Effective Pixels
2	Headphones	Quality	Comfort
3	Electric toothbrushes	Cleaning	Comfort
4	Humidifiers	Humidification	Quiet
5	Suitcases	Sturdiness	Durability
6	Projectors	Smoothness	Image
7	Robots	Obstacle Avoidance	Cleaning
8	Bicycles	Quality	Comfort

**Table 2:** The impact of three coding modes on attention.

Question: How Much Do the Alternatives Coded by xx Affect Your Attention (Paying More Attention to These Alternatives During the Decision Process)?						
	Impact Level	No	Low	Moderate	High	Very High
Coding Mode						
Color coding		16.92% (11)	24.62% (16)	18.46% (12)	36.92% (24)	3.08% (2)
Label coding		49.23% (32)	23.08% (15)	12.31% (8)	12.31% (8)	3.08% (2)
Size coding		13.85% (9)	21.54% (14)	33.85% (22)	23.08% (15)	7.69% (5)

**Table 3:** Preference degree of three coding modes.

Question: Please Rank the Three Coding Modes Used to Highlight Information on the Shopping Interface According to Your Preference.				
Coding Mode	Average Composite Score	1	2	3
Color coding	2.32	52.31% (34)	31.15% (19)	18.33% (11)
Label coding	1.57	16.92% (11)	31.15% (19)	51.67% (31)
Size coding	1.91	30.77% (20)	37.70% (23)	30.00% (18)

## Data Availability

Data will be available upon request.

## Abbreviations

The following abbreviations are used in this manuscript:

HCI	Human–computer interface
AI	Artificial intelligence
AR	Augmented reality
WITMED	Wise information technology of med
IM	Intelligent manufacturing
NPPs	Nuclear power plants
ECAM	Electronic centralized aircraft monitoring
ITS	Manned spacecraft and intelligent transport system
GP	Gaze plot
HM	Heat map
RFT	Relative fixation time
